# Rejection positivity predicts trial-to-trial reaction times in an auditory selective attention task: a computational analysis of inhibitory control

**DOI:** 10.3389/fnhum.2014.00585

**Published:** 2014-08-19

**Authors:** Sufen Chen, Robert D. Melara

**Affiliations:** ^1^Department of Neurology, Montefiore Medical CenterBronx, NY, USA; ^2^Department of Psychology, North Academic Center, City College, City University of New YorkNew York, NY, USA

**Keywords:** rejection positivity, selective attention, computational model, inhibitory control, distractor salience

## Abstract

A series of computer simulations using variants of a formal model of attention (Melara and Algom, 2003) probed the role of rejection positivity (RP), a slow-wave electroencephalographic (EEG) component, in the inhibitory control of distraction. Behavioral and EEG data were recorded as participants performed auditory selective attention tasks. Simulations that modulated processes of distractor inhibition accounted well for reaction-time (RT) performance, whereas those that modulated target excitation did not. A model that incorporated RP from actual EEG recordings in estimating distractor inhibition was superior in predicting changes in RT as a function of distractor salience across conditions. A model that additionally incorporated momentary fluctuations in EEG as the source of trial-to-trial variation in performance precisely predicted individual RTs within each condition. The results lend support to the linking proposition that RP controls the speed of responding to targets through the inhibitory control of distractors.

## Introduction

Identifying the neural mechanisms that enable flexible, goal-directed behavior has been a fundamental aim of cognitive neuroscience (Kerns et al., 2004; Friston, 2009). Yet establishing firm links between neural activity and its presumed cognitive or behavioral consequences has proved challenging. One reason is the ethical and technical limits (at least in humans) to manipulating behavioral responses from presumed physiological antecedents, relegating to merely correlational many linking hypotheses regarding behavioral outcomes (Teller, 1984; Schall, 2004). The ambiguity inherent in interpreting event-related potentials (ERPs), for example, hampers validation of linking propositions in cognitive tasks requiring selective attention (e.g., Hopf et al., 2004; Martinez et al., 2006; Müller et al., 2006). Here, ERP waves to attended signals typically evince greater voltage negativity than ERP waves to unattended signals, revealed in an ERP difference component called Nd (see Näätänen, 1990, for a review). As behavioral performance worsens with increasing task difficulty the magnitude of Nd shrinks and its onset latency lags. Thus, a specific physiological response (Nd magnitude) is associated with specific behavior outcomes (e.g., target reaction time). Yet here as elsewhere the psychological meaning of the neural activity is open to interpretation. Does Nd reflect increased attentional focus to targets? Does it reflect active inhibition of distractors? One goal of the present paper was to perform a formal computational analysis of ERPs during selective attention to evaluate more carefully than hitherto several hypothesized links between physiological activity and attentional processing.

### Rejection positivity

**Figure 1:**
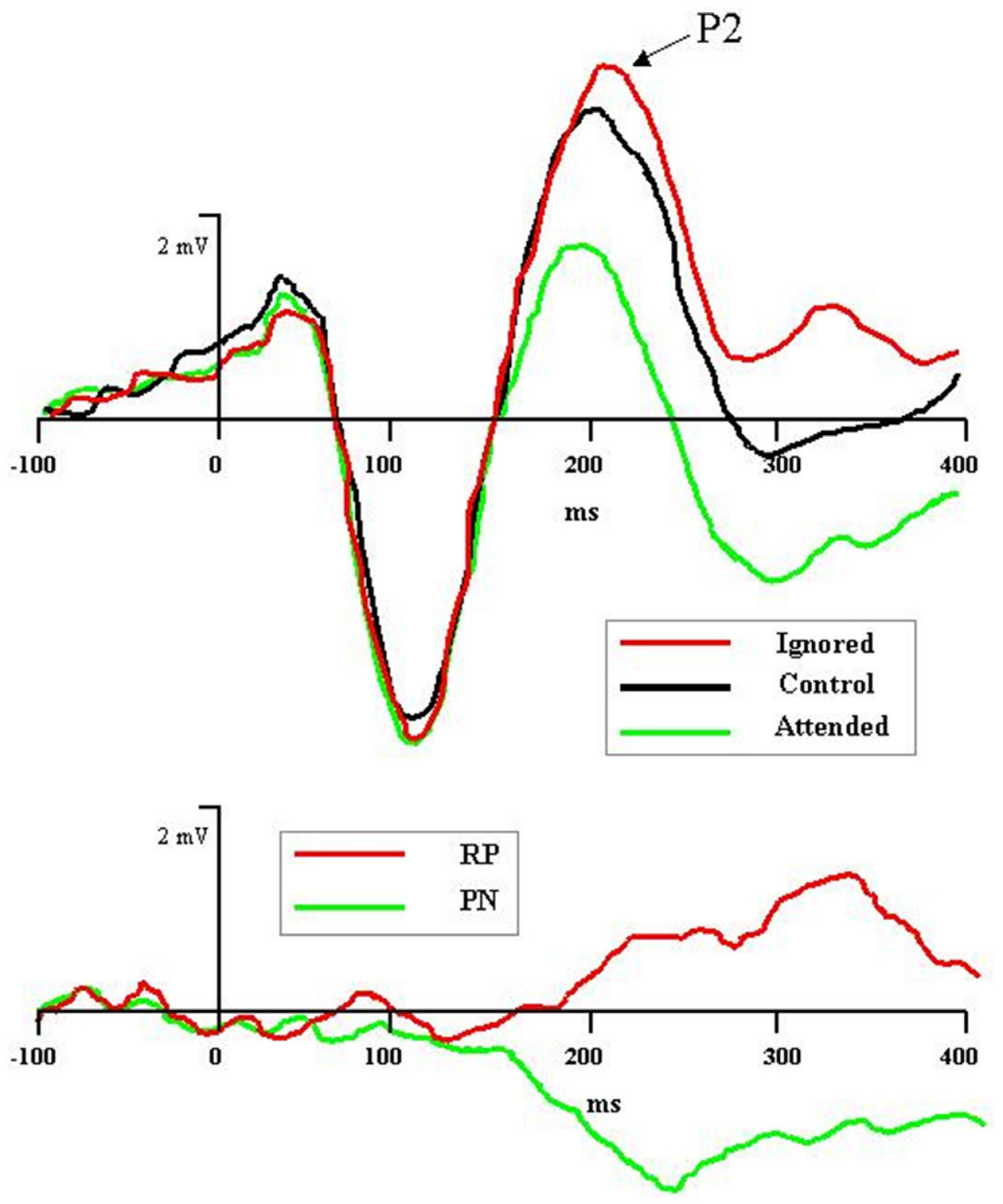
**Graphic depiction of rejection positivity (RP) to distractors and processing negativity (PN) to targets in an auditory selective attention task (after Bidet-Caulet et al., 2010). Top panel:** Grand averaged ERPs to attended (in green), control (i.e., non-attention, in black), and ignored (in red) stimuli reveal attention-induced separation of waveforms beginning with the P2 component, 200 ms after stimulus onset. **Bottom panel:** Difference of ignored vs. control waves (in red), depicting RP, and attended vs. control waves (in green), depicting PN.

Recent electrophysiological research has uncovered an ERP component specifically associated with the processing of distractors during tasks of selective attention (Münte et al., 2010; Mittag et al., 2013; cf. Power et al., 2012). The component—named rejection positivity (RP)—appears approximately 200 ms after distractor onset and can last 400 ms or more thereafter. Originally RP was identified in microscopic analyses of Nd: Relative to the ERP wave in a non-attention control condition, ERP waves to unattended stimuli in an attention task showed increased voltage positivity (Alho et al., 1987; Berman et al., 1989; Michie et al., 1990; Alain and Woods, 1994; Berman and Friedman, 1995). As depicted in Figure 1, rejection positivity thus served as the counterpoint to the processing negativity (PN) accompanying ERP waves to attended stimuli (Näätänen et al., 1978; Näätänen, 1982; Bidet-Caulet et al., 2010). Alho et al. (1987) offered the interpretation that the positivity reflected active suppression of rejected signals, hence the term rejection positivity. However, it also is possible that the ERP waves elicited in the so-called non-attention control condition are biased, either positively or negatively, thus leaving room for alternative explanations of RP (e.g., Michie et al., 1993; Alho et al., 1994).

Melara et al. (2002) were able to narrow the interpretive field of RP using results from an attention-training paradigm, which obviated the need for a non-attention baseline. Here, participants underwent 3 weeks of training to exercise skills of either auditory discrimination or distractor suppression, before and after being tested in discrimination and selective attention tasks. Thus, each participant served as his or her own baseline, against which RP could be revealed as the product of suppression training. Melara et al. found that suppression training, but not discrimination training, enhanced RP to distractors (but not PN to targets) beginning 200 ms after distractor onset. They further demonstrated that RP closely co-varied with improved behavioral performance during selective attention: The greater the positivity to distractors, the faster, less biased, and more accurate participants were in identifying targets. Since targets and distractors never overlapped in time, the authors suggested that RP regulates the salience of distractors held in working memory in the face of responses to currently perceived targets. More recently, Melara et al. (2012) showed that the effects of suppression training on RP peak several months after the final training session. The results of these studies suggest that RP is linked to a participant's ability to actively inhibit recent memories of distractors during selective attention to targets.

Other recent research has focused on the neural sources of RP. Bidet-Caulet et al. (2010) examined scalp topographies to attended and ignored ERP waves subtracted from non-attention baseline ERP waves (see Figure 1). The PN to attended tones had an earlier onset (150 ms) and a more anterior topography than the RP to ignored tones (200 ms). Nevertheless, the center-of-mass to both components was concentrated on frontal electrode sites. For RP, such activation may represent an executive control signal sourced in the frontal cortex, yet aimed at dampening activity in sensory cortex. In keeping with this interpretation, Chait et al. (2010), using MEG, recently found that distractor tones inserted temporally between two comparison tones elicited a magnetic RP component in the supra-temporal auditory cortex beginning 150 ms after distractor onset. The authors concluded that RP activity here reflected the sensory consequences of frontal inhibitory control mechanisms (see also Melara et al., 2002; Theeuwes and Chen, 2005).

### Manipulating distractor salience in working memory

In an investigation of monkeys trained to perform visual search, Ipata et al. (2006) reported slower and weaker activity to LIP neurons responsive to salient (popout) vs. non-salient distractors on days when the monkeys could successfully ignore the popout. Conversely, these LIP neurons were unusually active to popout distractors on days when the monkeys were unable to ignore them. The authors concluded that frontal control signals serve to suppress visual distractor activity on a parietal salience map (Gottlieb et al., 1998) thereby permitting easier target search (see also Bisley and Goldberg, 2003).

ERP evidence in humans indicates that RP is modulated by the salience of distractors in working memory. Melara et al. (2005) investigated salience by manipulating dimensional imbalance—that is, the psychophysical change along the distractor dimension relative to the target dimension (Melara and Mounts, 1993; Algom et al., 1996; Sabri et al., 2001). Here, targets and distractors never overlapped in time, ensuring that the influence of distractors on the processing of targets resided in memory. As the remembered salience of distractors increased from low to medium to high (across separate blocks of trials), participants' behavioral performance to perceived targets progressively deteriorated: They responded more slowly and committed more misidentifications of targets. These behavioral changes across conditions were accompanied by a monotonic reduction in RP occurring 400 to 600 ms after distractor onset. The authors concluded that as the salience of remembered distractors sharpened it became steadily more difficult for participants to inhibit in working memory, that is, which consequently undermined their task performance to targets.

As with many linking propositions, Melara et al.'s (2005) conclusion linking RP to inhibitory control of working memory rests solely on correlational evidence, that is, the finding that differences in levels of salience were associated both with corresponding changes in neural activity (RP magnitude) and behavior (RT and accuracy). However, these associations themselves spring largely from the joint effects created across the different salience conditions. Thus, the authors' attribution of inhibitory control was merely inferred from group averages. An alternative interpretation is that RP gauges perceived stimulus salience but plays no causal part in managing attentional control. The goal of the present study was to explore more precisely the possible role of RP in the inhibitory control of distraction in memory using a computational model that permitted microscopic (trial-to-trial) analyses of the connection between physiology and behavior.

### Model-based connections between neural activities and behavioral performance

Model-based cognitive neuroscience represents a powerful new approach to evaluate linking hypotheses (Gold and Shadlen, 2000, 2003; Forstmann et al., 2011). Here, cognitive constructs are formalized mathematically, connecting brain activation computationally with behavioral outcomes (e.g., Holroyd and Coles, 2002; Roitman and Shadlen, 2002; Wang, 2002; Sugrue et al., 2004; Purcell et al., 2010; van Maanen et al., 2011). Purcell et al. (2010), for example, formalized the construct of (target and distractor) evidence accumulation, which mathematically linked spike activity in monkey frontal eye fields (FEF) to saccadic latency during visual search. Variants on the model distinguished the form of evidence accumulation (e.g., perfect, leaky, gated, etc.); goodness of fits between observed and predicted RT distributions successfully discriminated among model variants. The analysis supported the authors' linking proposition that neurons in FEF accumulate perceptual evidence until threshold, triggering saccadic movement toward target or distractor locations.

**Figure 2:**
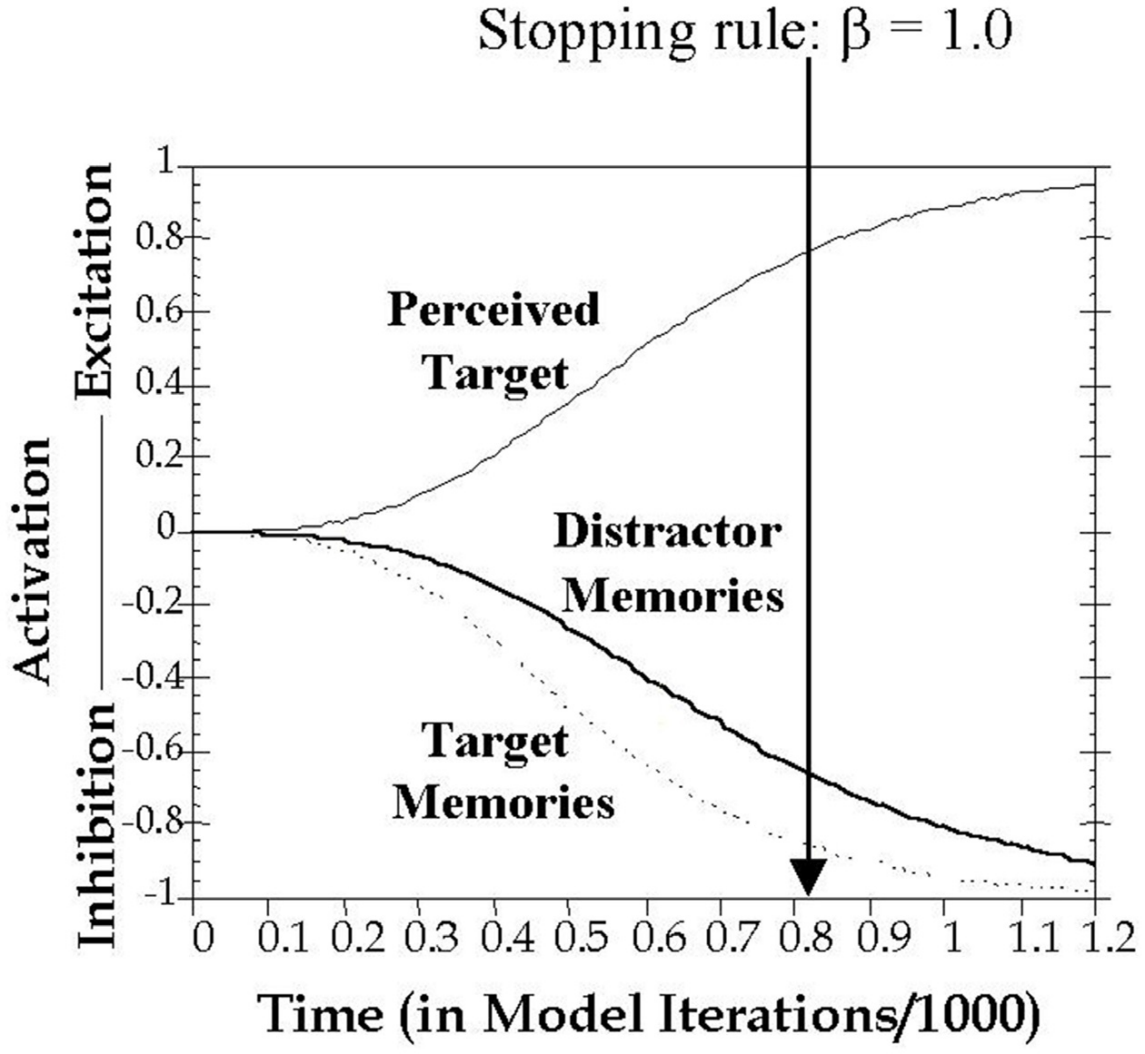
**Graphic depiction of the time course of activation on a filtering trial.** Presentation of a target stimulus simultaneously triggered excitation of the target's perceptual representation and inhibition of memory representations of previous targets and distractors. The time to reach threshold is classification RT, modeled as the number of cycles *t* needed to satisfy the stopping rule, which was set in all simulations here to β = 1.0. Imagine moving the vertical threshold line progressively from left to right until β = 1.0. Where the line stops (β = 1.0) is the predicted RT on the trial.

The current study formalizes inhibitory control in working memory using the computational model proposed by Melara and Algom (2003). As shown in Figure 2, here attentional selection is conceived as a dual process, concurrently involving excitation of the current target and inhibition of all trial-irrelevant information, including all previous distractors held in memory. Distractor salience (among other factors) influences the buildup of excitation to the current target and inhibition to distracting information. A decision variable connects target choice to a predefined stopping rule of the ratio of activations between target and non-target stimulus values (see Method for details). Thus, the speed of responding is intrinsically linked in the model to the rate of growth in excitatory and inhibitory activity.

### The current modeling study

In the original version of the model, Melara and Algom (2003) used free mathematical parameters to estimate the rates of excitatory and inhibitory activity. However, in the current study, we used a brain variant of the model to evaluate the linking proposition that RP is associated as salience grows with the loss of inhibitory activation to distractors. Here, recorded values of RP substituted for the free parameters in determining the rates of activation to distractors. Inhibitory activation thence contributes to the weight of evidence (Gold and Shadlen, 2007) the perceiver considers about how to respond to the target stimulus. In this way, the model connects RP to behavioral decisions through the construct of inhibitory activation. We asked whether an alternative model in which recorded values of PN were used to set excitatory activation of targets could equally predict the effects of distractor salience on behavioral performance. Thus, we ask, does RP or PN better explain the variance in behavioral effects from distractor salience?

We sought in the current study to predict behavioral responses from physiological data at both the condition level and the individual-trial level. Our behavioral outcome variable was reaction time (RT). At the condition level, we used RP (and PN) to simulate overall behavioral performance in each condition. At the individual-trial level, we used momentary fluctuations in the low-frequency oscillations to distractors that give rise to RP (i.e., RP calculated from single trial EEG, hereafter, *RP-noise*) to simulate the RT of each participant on each trial. Our modeling efforts rest on the assumption that RP-noise is not merely random variation in the bioelectric signal, but is in fact meaningfully associated with stochastic behavioral processes. We evaluated this assumption by comparing model simulations that predict behavioral variation using RP-noise with those using random noise drawn from either Gaussian (hereafter, *Gaussian-noise*) or rectangular (hereafter, *Rectangular-noise*) distributions. Our final version of the model combined EEG-based estimates of inhibitory activity with RP-noise to examine how well a fully linked neurobehavioral model could predict the individual RTs on every trial of every participant in every condition.

## Methods

### Data source

**Figure 3:**
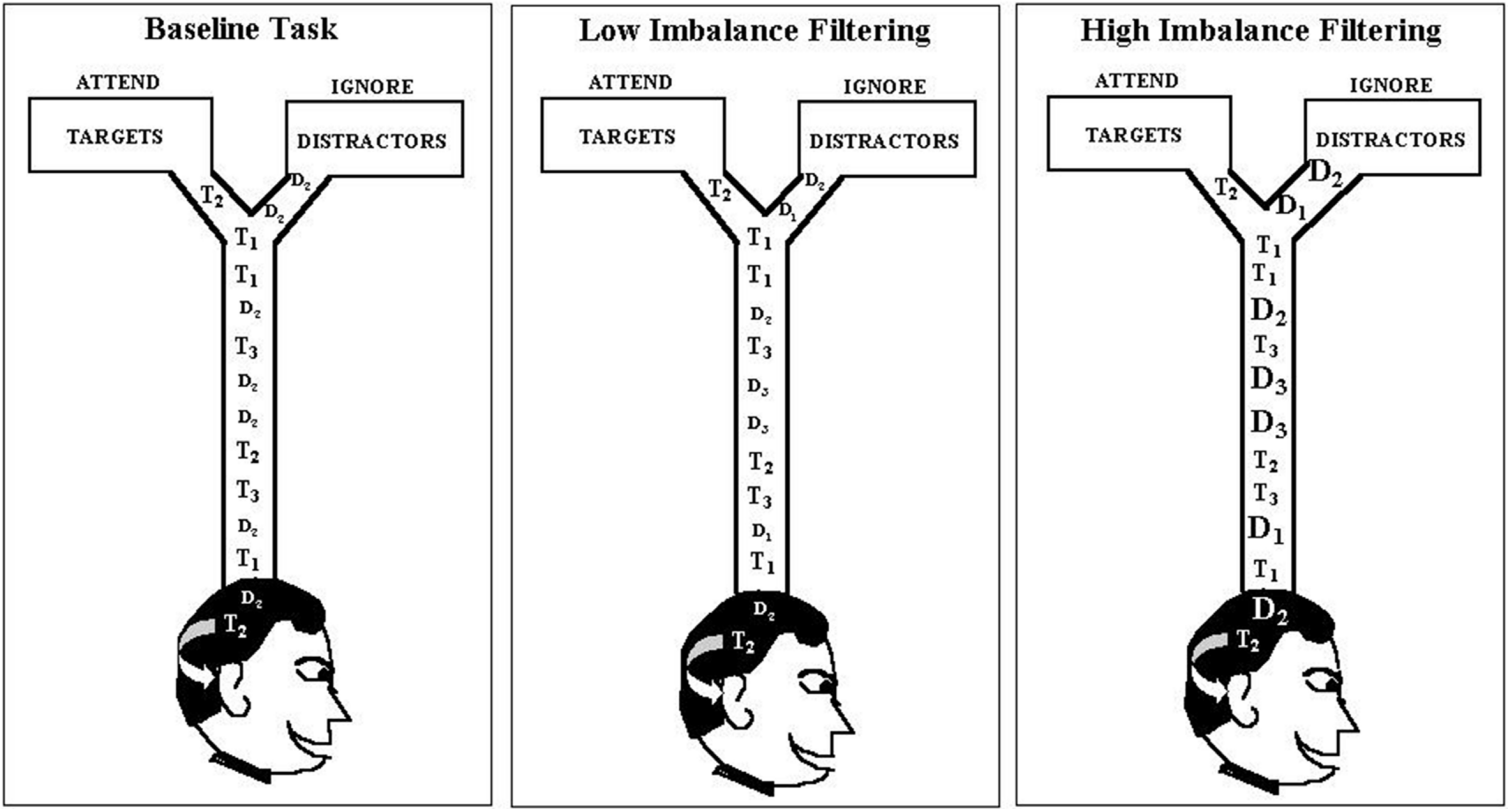
**Graphic depiction of the modified Garner paradigm used as the data source in the current modeling study.** Each filtering task contained three distractors, with the middle distractor (D_2_) the same as in the baseline task (1020 Hz), but the pitch range (marked by D_1_ and D_3_) increasing progressively from the low-imbalance filtering task (Filtering 1: D_1_ = 982 Hz, D_3_ = 1060 Hz) to the high-imbalance filtering task (Filtering 3: D_1_ = 929 Hz, D_3_ = 1120 Hz). Note: Filtering 2 (i.e., medium-imbalance task) is absent in the depiction.

The empirical data used in our simulations were reported as Experiment 1 in Melara et al. (2005). Eleven participants were tested. Stimuli were rectangular-wave tones in the 1-kHz range, 100 ms in duration (10 ms rise/fall) presented binaurally at 73 dB, digitized to 16 bits at a sampling rate of 48 kHz. The stimulus set, which appears in Table 1, was used to construct one baseline condition (single distractor) and three filtering conditions (multiple distractors) of the so-called Garner paradigm (Garner and Felfoldy, 1970; Garner, 1974; see Figure 3). Each condition included three targets of differing auditory frequency (962 Hz, 1000 Hz, and 1040 Hz); targets were the same across the four conditions. In the baseline condition, the distractor was always 1020 Hz. In each filtering condition, the distractor set contained three tones, the middle one matching the baseline distractor. The low and high tones in the distractor set differed increasingly in within-channel discriminability (physical distance) across the three filtering conditions, as summarized in Table 1, creating low (Filtering 1), medium (Filtering 2), and high (Filtering 3) imbalance relative to the target set. Differences between targets and distractors were defined by the timbre of the tones, targets having a duty cycle of 36%, distractors 18%. Each condition contained 300 trials, 150 target trials (50 trials per target stimulus, *P* = 0.17) and 150 distractor trials (50 trials per distractor stimulus in filtering, *P* = 0.17, 150 in baseline, *P* = 0.50). Stimuli were selected at random from the target or distractor set; targets never appeared together in time with distractors. The onset-to-onset interval between any two stimuli ranged randomly between 1450 ms and 1600 ms in rectangular distribution. The task requirements were identical in each condition: identify each stimulus to be one of three targets by pressing one of three keys on a keyboard (designated low, medium, and high) with the index, middle, and ring fingers of the dominant hand, while ignoring and withholding responses to all distractors. Participants listened for the frequency differences among targets and the timbre differences between targets and distractors using an interactive display presented before each block of trials. Each participant was asked to respond to targets as quickly as possible while maintaining accuracy. Response keys were counterbalanced across participants. EEG recordings were time-locked to each stimulus (targets and distractors); behavioral performance (RT and accuracy) was measured to each target. We only simulated target trials involving a correct response (range from 317–568 trials across participants, with an average of 474 correct trials in any condition for each participant).

**Table 1:** **Auditory frequencies (in Hz) in each of four conditions (Baseline, Filtering 1, Filtering 2, and Filtering 3)**.

**Condition**	**Target 1**	**Target 2**	**Target 3**	**Distractor 1**	**Distractor 2**	**Distractor 3**
Baseline	962	1000	1040	1020	1020	1020
Filtering 1	962	1000	1040	982	1020	1060
Filtering 2	962	1000	1040	954	1020	1090
Filtering 3	962	1000	1040	929	1020	1120

**Figure 4:**
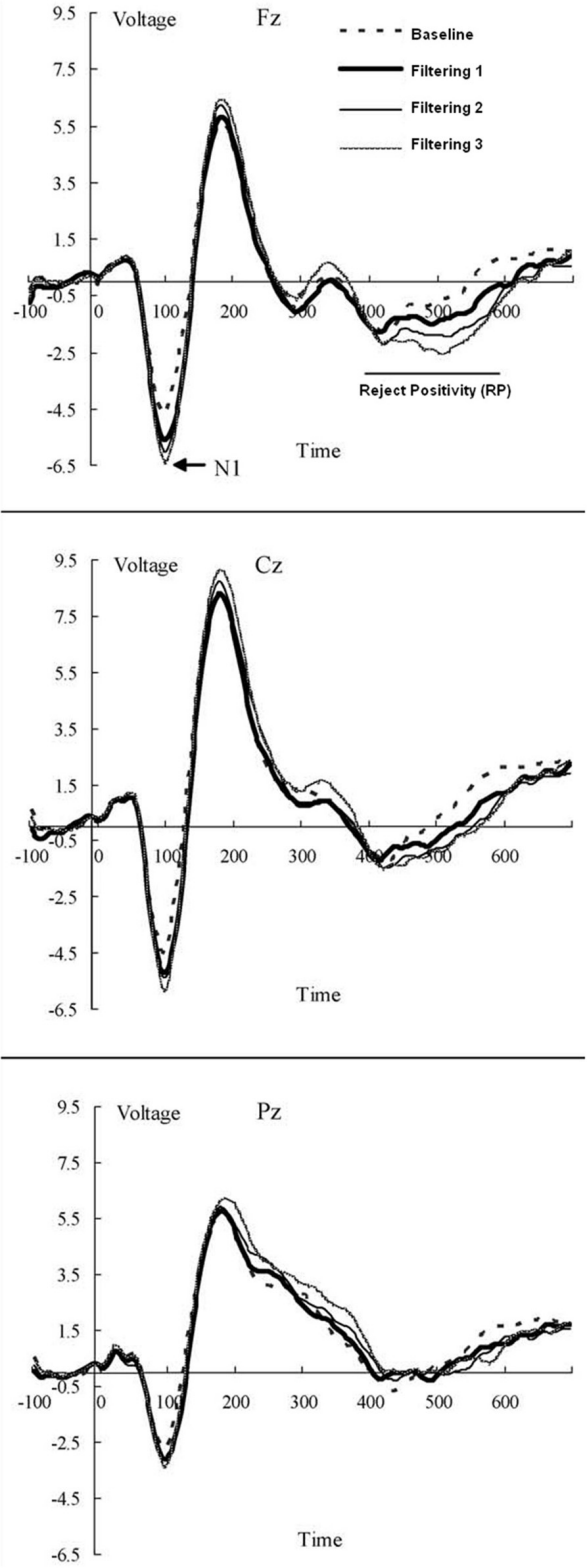
**Grand-averaged waveforms (in AV) of ERPs from midline electrode sites (Fz, Cz, and Pz) elicited by distractors in each of the four conditions (baseline; low, medium, and high filtering) of the target-constant tasks in the source data.** Increased distractor discriminability reduced RP: a frontal inhibitory slow-wave component (400–600 ms after stimulus onset).

The EEG was recorded from 13 scalp locations (Fz, F3, F4, Cz, C3, C4, Pz, P3, P4, T3, T4, T5, and T6 of the International 10–20 System) with tin electrodes mounted in a stretch cap (Electro-Cap International). Only the Fz site was used in modeling. The electrodes were referenced to linked mastoids (LM and RM) off-line with the Fpz as the ground electrode. Impedance was maintained below 2 kΩ across all sites. Blinks and other eye movements were monitored by electrooculogram (EOG) from two electrode montages, one on the infra- and supra-orbital ridges of the left eye (VEOG), the other on the outer canthi of each eye (HEOG). EEG and EOG signals were analog filtered with a band pass from 0.1 to 100 Hz (–3 dB cutoffs) and digitized at 250 Hz. Trials containing EEG or EOG activity exceeding 100 μV was rejected as artifacts. The EEG was averaged for each condition from 100 ms before the stimulus to 700 ms post-stimulus, using the 100 ms pre-fixation period as baseline, with RP amplitude calculated as the mean voltage in the 400–600 ms post stimulus window at Fz (Figure 4).

### Activation function to the currently presented target

Our overall approach to theorizing and model fitting followed Melara and Algom (2003). As depicted in Figure 2, we assumed, as they did, that presentation of a target stimulus simultaneously triggers activation of the current target's representation, as well as representations of targets from previous trials, and representations of distractors from previous trials. The perceived target on a trial receives excitatory processing with the activation value increasing until asymptote or until a decision is made:

(1) $$\text{Act}({\text{T}}_{\text{i}},\text{t})=\frac{1}{{\left[1+\exp(-{\text{slope}}_{{\text{T}}_{\text{i}}}*\text{t})\right]}^{10}},$$

where the inverse logistic function is raised to a scaling constant of 10, T_i_ is the stimulus value on a target trial (*i* = 1, 2 or 3, with one of three possible targets appearing on each target trial in each condition), slope_T_i__ is the slope of activation for T_i_, and Act(T_i_,t) is the activation value for T_i_ at time *t*.

### Activation function to non-presented targets on target trials

In simulating the target trials, each non-presented stimulus, including both distractor and non-presented targets, is inhibited from target onset. The inhibitory activation value to currently non-presented targets in the stimulus set (T_j_, j = 1… 3 and j ≠ i) decreases until asymptote or until a decision is made:

(2) $$\text{Act}({\text{T}}_{\text{j}},\text{t})=\frac{-1}{{\left[1+\exp(-{\text{slope}}_{{\text{T}}_{\text{j}}}*\text{t})\right]}^{10}}$$

where slope_T_j__ is the slope of the activation function to currently non-presented target T_j_ and Act(T_j_,t) is the activation value for T_j_ at time *t*. Unlike Melara and Algom (2003), we constrained the slope of T_j_—a target value not currently presented—to equal the slope of the same target on trials in which it had actually been presented (i.e., T_i_). Thus, the activation functions in these two situations (i.e., perceived and remembered) differ only in direction of activation, that is, increasing (toward an asymptotic bound of +1) for perceived targets (Equation 1), reflecting an excitatory process, and decreasing (toward a bound of −1) for remembered targets (Equation 2), reflecting an inhibitory process. This simplifying assumption reduced free parameters while minimally affecting model fits.

### Activation function to distractors on target trials

As in the case of non-presented targets, we modeled distractor processing as inhibitory activation from the onset of each target trial. However, to better equate comparisons with target processing, here we estimated a single inhibitory slope^1^ to the three different distractors on each target trial, which elevated the inhibitory bound of activation to −3:

(3) $$\text{Act}({\text{D}}_{\text{s}},\text{t})=\frac{-3}{{\left[1+\exp(-{\text{slope}}_{\text{d}}*\text{t})\right]}^{10}}$$

where slope_d_ is the average slope of inhibitory activation across the three distractors and Act(D_s_,t) is the distractor activation value on a target trial at time *t*.

### Transformation of activation into weight of evidence

The decision variable about how to respond to the presented target is a weight of evidence derived from the log ratio of two types of activation: The ratio's numerator contains evidence favoring the perceived target, the denominator evidence against the perceived target which, in the current study, amounts to transformed activations of remembered (currently non-presented) targets and distractors. Suppose that target T_i_ appears on the current trial. We assume that the representation for T_i_ is activated in the form of excitation, and that the representations for all the other stimuli in memory are inhibited. The momentary goal of the decision mechanism is to determine whether the weight of evidence favoring T_i_ meets a preset stopping rule β at each time *t*. The goal is achieved by a decision variable that integrates the excitatory evidence for T_i_ with evidence against T_i_ supplied by inhibitory activation values from the other stimuli in the set:

(4) $$\begin{array}{l} \text{Evidence}({\text{T}}_{\text{i}},\text{t})=\text{In}\frac{\text{Act}({\text{T}}_{\text{i}},\text{t})+\text{constant}}{\sum_{\text{j}}\text{Act}({\text{T}}_{\text{j}},\text{t})+\text{Act}({\text{D}}_{\text{s}},\text{t})+\text{constant}},\\ \;(\text{j}=1\ldots \;3,\text{ and j}\neq \text{i}) \end{array}$$

where Act(T_i_,t) is the activation value for the currently presented target T_i_ at time *t* (calculated according to Equation 1), Act(T_j_,t) is the activation value for each currently non-presented target T_j_ (j = 1 … 3, and j ≠ i) at time *t* (Equation 2), and Act(D_s_,t) is the activation value for the currently non-presented distractors at time *t* (Equation 3). (*constant* = 6.0 for all simulations to scale evidence within the range of the stopping rule). Expanding the evidence function into its three different terms (i.e., Equations 1–3):

(5) $$\begin{array}{l} \text{Evidence}({\text{T}}_{\text{j}},\text{t})=\text{In}\frac{\frac{1}{{\left[1+\exp(-{\text{slope}}_{{\text{T}}_{\text{i}}}*\text{t})\right]}^{10}}+6}{\sum_{\text{j}}\frac{-1}{{\left[1+\text{exp}(-{\text{slope}}_{{\text{T}}_{\text{j}}}*\text{t})\right]}^{10}}+\frac{-3}{{\left[1+\exp(-{\text{slope}}_{\text{d}}*\text{t})\right]}^{10}}+6}\\ \;(\text{j}=1\ldots \;3,\text{ and j}\neq \text{i}) \end{array}$$

A decision is made and a response emitted once Evidence (T_i_,t) reaches the decision threshold β. The time it takes to reach the threshold is the classification RT, modeled in the present study as the number of cycles *t* (repeated iterations of Equation 5 with increasing values of *t*) needed to reach the decision threshold. For all simulations in the present study, the stopping rule was set to β = 1.0 (see Figure 2).

### Model-fitting procedure

For each simulated target trial *j* four slopes (slope_T1_, slope_T2_, slope_T3_, and slope_d_) are needed to calculate the decision variable for each participant (see Equation 5). The equations used to obtain these slopes were modifications of those used by Melara and Algom (2003):

(6) $$\begin{array}{l} {\text{slope}}_{{\text{T}}_{\text{i}}}[\text{j}]=\frac{1}{1+\exp(-({\text{tstart}}_{{\text{T}}_{\text{i}}}+\text{tswell}*\text{x}))}*0.023\\ \;(\text{i}=1,\;2,\text{ or }3) \end{array}$$

(7) $${\text{slope}}_{\text{d}}[\text{j}]=\frac{1}{1+\exp(-(\text{dstart+dswell}*\text{x}))}*0.023$$

where the logistic function scales the slope and *tstart*_T_i__ (*i* = 1, 2, or 3), *tswell*, *dstart*, and *dswell* are mathematical parameters modulating the rate of exponential growth in slope as a function of stochastic variability, estimated in the current study either from the model-fitting procedure or from actual EEG data. By controlling the rate of target and distractor activation these parameters served as the primary basis of differences among the excitatory and inhibitory models explored in the current study. The multiplicative constant of 0.023 replaced two additional free parameters in Melara and Algom, thus simplifying the basic model. *x* is the value of a random variable denoting noise on trial *j*. Changes in the value of *x* from one trial to the next were the basis of trial-to-trial variability in RTs, yielding a predicted RT on each individual trial. In simulations using random noise, values of *x* were generated from a Gaussian or a rectangular distribution. In simulations using RP-noise, values of *x* were obtained from EEG data as the average voltage 400–600 ms after stimulus onset (Figure 4), individually for each artifact- and response-free distractor trial.

We performed model fitting in each simulation using first a parameter-estimation stage and then a backfit solution stage. The mathematical goal of the parameter-estimation stage was to minimize the following objective function:

(8) $$\text{objFunc}=\sum_{\text{h}=1}^{\text{N}}\left[(\beta -\text{Evidence }{\left({\text{T}}_{\text{i}}(\text{h}),\text{ t}\right)}^{2}\right]$$

where Evidence (T_i_,t) is defined as in Equation 5, yet substituting the observed RTs for *t*. T_i_ is the target on the *h*th trial, denoted as T_i_(h), β = 1.0, *t* is the observed response time on the *h*th trial, and N is the number of trials in a condition. Parameter estimates were defined as those values of *tstart*_T_i__ (*i* = 1, 2, or 3), *tswell*, *dstart*, and *dswell* that minimized Equation 8 for a given participant within a given condition. The minimization procedure for parameter estimation used the Downhill Simplex method of Nelder and Mead (1965; see also Press et al., 1992; Vetterling et al., 1992; Gershenfeld, 1999). In a subsequent backfit solution stage, the parameter estimates derived in the earlier stage were inserted into Equations 6 and 7 with *t* as an unknown, so that the simultaneous system of equations could then be solved trial-by-trial for *t* in Equation 8. The value of *t* on trial *h* that minimized objFunc served as the predicted RT to target T_i_ on that trial.

### Model evaluations

We used two techniques to evaluate model fit: group RT distributions and distributional moments. First, to evaluate each simulation at the condition level, we compared the averaged observed vs. predicted RT distributions. The technique resembles that devised by Vincent (1912) for viewing learning curves and so is often referred to as Vincent's procedure or Vincentizing (Ratcliff, 1979). In the current analysis, each participant's RTs were first sorted in ascending order and then divided into 20 quantiles, such that the first 5% of RTs comprised the first quantile, the second 5% the second quantile, and so on. RTs were then calculated by separately averaging RTs in each of 20 individual quantiles for each participant in each condition. A group distribution for each condition was obtained by averaging RTs across participants in each quantile. An analogous group distribution was derived from the predicted RTs in each simulation. To highlight differences among simulations, we depicted the group RT probability density functions as smooth line graphs with each quantile probability calculated as:

(9) $$\text{P}(\text{i})=\frac{50}{\text{RT}(\text{i}+1)-\text{RT}(\text{i})}\;(\text{i}=1,2,\ldots n-1)$$

where P(i) is the probability of the *i*th group quantile, RT(i) is the quantile RT for the *i*th group quantile (in ms), and *n* is the number of quantiles. To evaluate the goodness-of-fit of each simulation statistically, we compared the observed vs. predicted group distributions using chi-square analysis.

Second, to assess how closely predicted and observed RTs corresponded at the level of individual trials, we examined the first four moments (mean, variance, skewness and kurtosis) of the individual participant (unaveraged) RT distributions. Skewness and kurtosis were calculated using the following equations (Cramer, 1946):

$$\begin{array}{l} \text{skewness}=\frac{\sum_{\text{i}=1}^{\text{n}}{\left(\text{RTi}-\text{mean}\right)}^{3}}{n*\delta^{3}}\\ \text{kurtosis}=\frac{\sum_{\text{i}=1}^{\text{n}}{\left(\text{RTi}-\text{mean}\right)}^{4}}{n*\delta^{4}}-3 \end{array}$$

where *n* is the number of trials in the condition, RT_i_ is the reaction time for the *i*th trial, *mean* is the average RT across the *n* trials, and δ is square root of the RT variance in the condition. For each simulation, we conducted a repeated-measures analysis of variance (ANOVA) on moment values, with Moment (4 levels; mean, variance, skewness and kurtosis), Simulation (2 levels: observed vs. predicted), and Condition (4 levels: baseline, filtering 1, 2, and 3) as within-subject factors. Statistically significant main effects or interactions involving Simulation indicate poor model fit. The analysis of distributional moments provides finer granularity and greater rigor in model evaluation than is possible when examining sets of correlation coefficients between observed and predicted RTs, where a separate coefficient (from ~475 data points) is needed for each participant in each condition. The microanalysis of moments complemented the condition-level analysis of group distributions by isolating in each model the four central sources of trial-to-trial change in behavioral performance (Sternberg, 1969a,b; Ratcliff, 1979).

## Results

### Excitatory activation vs. inhibitory activation in the face of distraction

The aim of the first pair of computer simulations was to compare fits of an excitation-only model to fits of an inhibition-only model as a means of assessing whether participants primarily coped with the increasing distractor salience across conditions by varying excitation to the targets or by modulating inhibition of the distractors. Each model assumes that both short- and long-term memory processes determine rates of activation to target or distractor representations. The current paper evaluates the role of long-term memory in selective attention failure. Roughly, the start parameters (tstart in Equation 6, dstart in Equation 7) reflect the influence of long-term memory on target or distractor activations, respectively, whereas the swell parameters (tswell and dswell) describe the interaction of short- and long-term memory, with x reflecting momentary fluctuations in short-term memory. The excitation-only model emphasizes the relative influence on performance of target activations in long-term memory, whereas the inhibition-only model emphasizes executive control of distractor activations in long-term memory.

We began each simulation by estimating a set of six free parameters in the baseline (i.e., single distractor) condition: four parameters (i.e., tstart_T1_, tstart_T2_, tstart_T3_, and tswell in Equation 6) controlled the excitation of the targets and two parameters (i.e., dstart and dswell in Equation 7) the inhibition of the distractors. In the inhibition-only version, the distractor parameter dstart was estimated separately in each of the four conditions, whereas the estimates made at baseline to the remaining five parameters (i.e., dswell, tstart_T1_, tstart_T2_, tstart_T3_, and tswell) were applied to each of the three filtering (i.e., multiple distractor) conditions. The inhibition-only model thus required 9 free parameters. In the excitation-only version, the tstart parameter to each target (T_1_, T_2_, and T_3_) was estimated separately in each condition, whereas baseline estimates of the remaining three parameters (i.e., dstart, dswell, and tswell) were used in each of the three filtering conditions. The excitation-only model required 15 free parameters. In both models both inhibitory and excitatory processes were activated on each trial.

**Figure 5:**
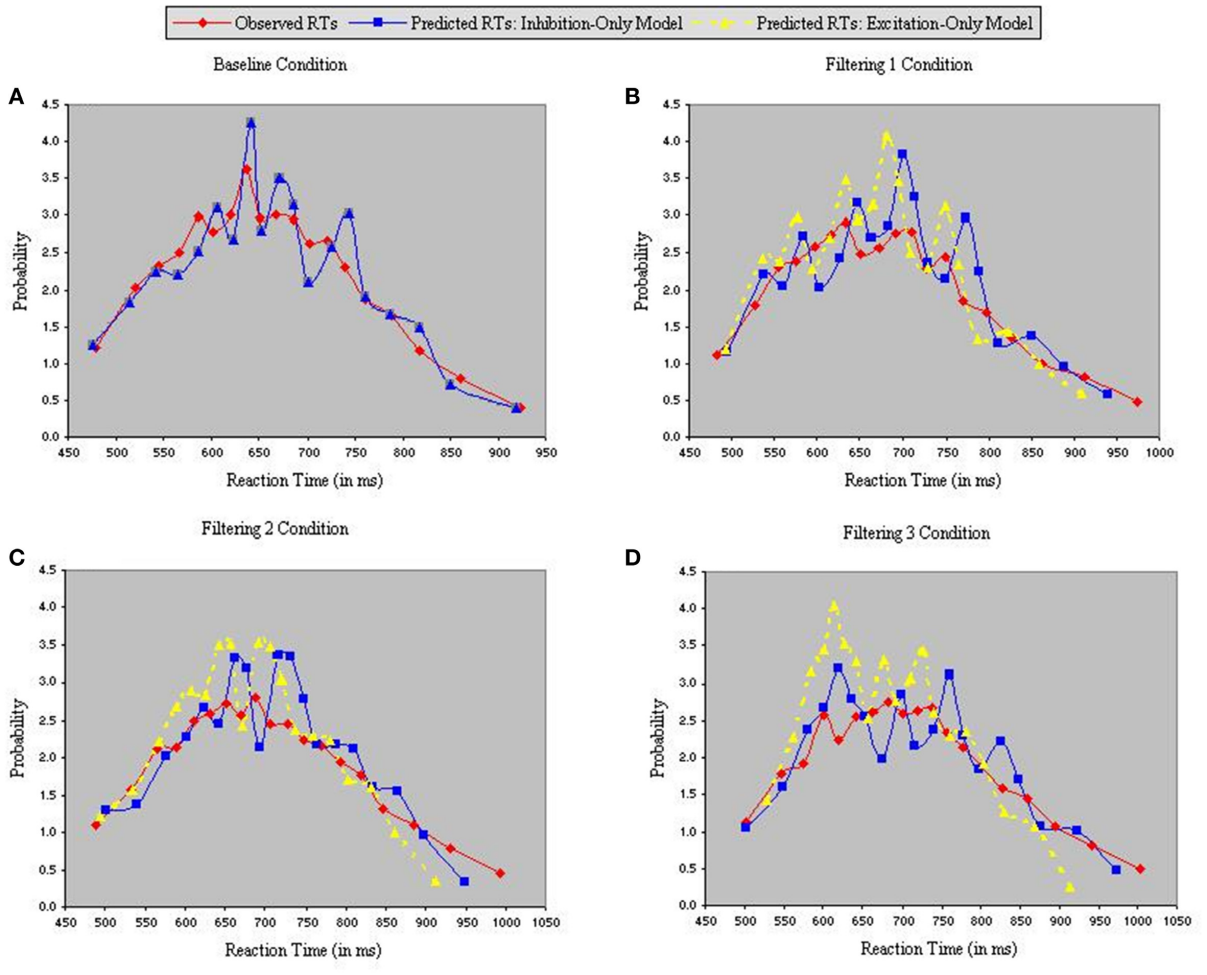
**Probability density functions of observed RTs together with predicted RTs for the Inhibition-Only Model (Gaussian noise) and the Excitation-Only Model (Gaussian noise), each panel representing one of four conditions (A = Baseline, B = Filtering 1, C = Filtering 2, and D = Filtering 3)**.

Figure 5 depicts density functions of the observed and predicted RTs of the inhibition-only and excitation-only models, with each panel illustrating a different condition. Chi-square tests were performed between observed and predicted RTs in each condition to evaluate the goodness-of-fit of each model. The two models are identical in the baseline condition, so produced equally good fits in Figure 5A [χ^2^_(17)_ = 4.25]. However, fits of the two models diverged in the three filtering conditions (Figures 5B–D), with the analysis of predicted RTs in the excitation-only model revealing relatively poor fit in each condition [Filtering 1, χ^2(17)^ = 21.35; Filtering 2, χ^2^_(17)_ = 26.79; Filtering 3, χ^2^_(17)_ = 23.44]. By contrast, predicted RTs of the inhibition-only model, notwithstanding its 6 fewer parameters, more closely corresponded to the observed RTs [Filtering 1, χ^2^_(17)_ = 10.06; Filtering 2, χ^2^_(17)_ = 9.13; Filtering 3, χ^2^_(17)_ = 5.41].

Further insight is gained from an analysis of distributional moments. Table 2 summarizes the four distributional parameters (mean, variance, skewness and kurtosis) of the observed RTs in each condition. Table 3 tallies the differences between observed and predicted moments for each model. As one can see, the excitation-only model underestimated in each filtering condition both the mean RT and the RT variance (mean RT underestimated by 20 ms, 26 ms, and 24 ms in the Filtering 1, Filtering 2, and Filtering 3 conditions; RT variance by 25 ms, 22 ms, and 26 ms). RT predictions of the inhibition-only model led to slightly compressed estimates of variance, but only in the Filtering 1 and 2 conditions; otherwise the model more closely mimicked all four distributional moments. Overall, despite its 6 fewer parameters, the inhibition-only model edged the excitation-only model in capturing distributional characteristics of each participant in each condition. Nevertheless, ANOVA indicated that neither model yielded exceptional fits to the observed data, with both simulations evincing statistically significant differences between observed and predicted moment values [*Excitation-Only*: Simulation, *F*_(1, 10)_ = 36.1, *p* < 0.001; Simulation × Condition, *F*_(3, 30)_ = 15.07, *p* < 0.001; Simulation × Moment, *F*_(3, 30)_ = 30.57, *p* < 0.001; Simulation × Condition × Moment, *F*_(9, 90)_ = 12.21, *p* < 0.001; *Inhibition-Only*: Simulation, *F*_(1, 10)_ = 9.34, *p* < 0.01; Simulation × Condition, *F*_(3, 30)_ = 6.5, *p* < 0.001; Simulation × Moment, *F*_(3, 30)_ = 11.36, *p* < 0.001; Simulation × Condition × Moment, *F*_(9, 90)_ = 7.12, *p* < 0.001].

**Table 2:** **The first four distributional moments (mean, variance, skewness, and kurtosis) of the observed RT in each of four conditions (Baseline, Filtering 1, Filtering 2, and Filtering 3)**.

**Condition**	**Mean**	**Variance**	**Skewness**	**Kurtosis**
Baseline	696.761	141.345	0.833	0.734
Filtering 1	720.994	153.733	0.704	0.283
Filtering 2	738.285	157.574	0.628	0.112
Filtering 3	748.382	155.825	0.612	0.040

**Table 3:** **Difference between predicted and observed moments in RTs (mean, variance, skewness, and kurtosis) for the Inhibit ion-Only Model and the Excitation-Only Model**.

**Condition**	**Mean**	**Variance**	**Skewness**	**Kurtosis**
**INHIBITION-ONLY MODEL (GAUSSIAN NOISE)**				
Baseline	−0.164	−0.543	−0.012	0.282
Filtering 1	−1.884	−16.056	−0.176	−0.253
Filtering 2	−1.717	−12.208	0.102	0.688
Filtering 3	0.365	−2.351	−0.017	0.088
**EXCITATION-ONLY MODEL (GAUSSIAN NOISE)**				
Baseline	−0.164	−0.543	−0.012	0.282
Filtering 1	−19.732	−25.176	−0.111	−0.137
Filtering 2	−25.608	−22.109	0.170	0.877
Filtering 3	−24.014	−26.100	0.073	0.491

### Rejection positivity vs. processing negativity

The aim of the second pair of simulations was to evaluate the linking hypothesis that RP is a biomarker of inhibitory control during tasks of selective attention, ultimately affecting the speed to decide target identity. To achieve this goal, we estimated slope_d_ in each condition using actual RP amplitudes. Parameter estimation for the RP model began with a linear regression in which the estimates of *dstart* from the four conditions of the inhibition-only simulation were used to predict slow-wave amplitude of each participant's averaged ERP waves at Fz, 400–600 ms after distractor onset (see Melara et al., 2005). By estimating only the regression coefficient and the Y intercept we were able to reduce the total number of free parameters of the inhibition-only model from 9 to 7. For example, a participant's *dstart* in the Filtering 1 condition equaled the Y intercept added to the product of the regression coefficient and the average RP amplitude in that condition. After obtaining *dstart* values, the *tstart*, *tswell*, and *dswell* parameters of Equations 6 and 7 were re-estimated in the baseline condition using Downhill Simplex to obtain the best baseline fit. As comparison, we simulated a PN model in which slope_T1_, slope_T2_, and slope_T3_ in each condition were estimated using magnitudes of target PN, that is, each participant's slow-wave ERP amplitude to the target at Fz, 400–600 ms after target onset. We used a linear regression method analogous to that used in the RP model to estimate the *tstart* parameters in each condition, which reduced the total number of free parameters of the excitation-only model from 15 to 9. This approach allowed us to evaluate a neurobehavioral model of inhibition-only (RP model) directly against a neurobehavioral model of excitation-only (PN model).

**Figure 6:**
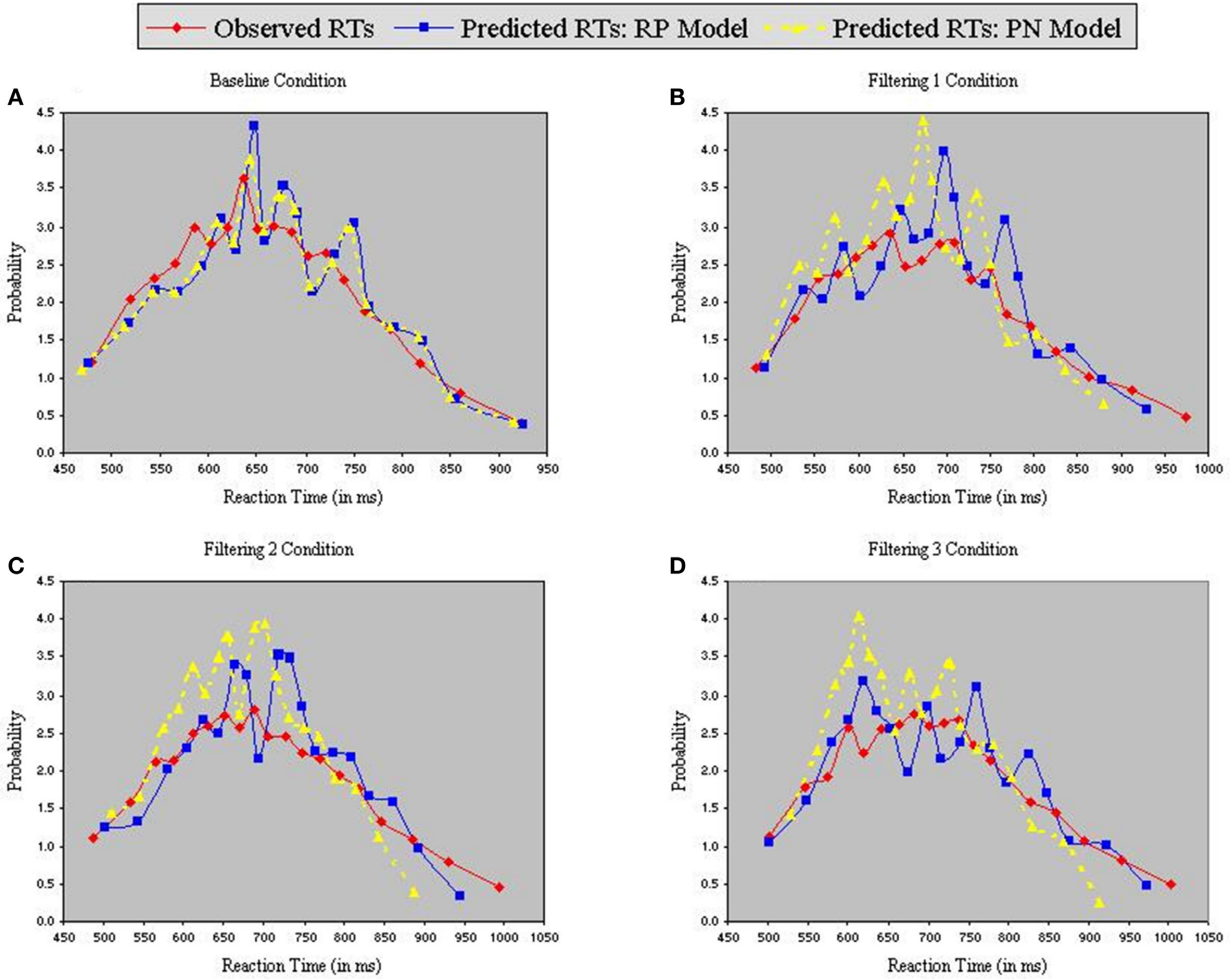
**Probability density functions of observed RTs together with predicted RTs for the RP Model (Gaussian noise) and the PN Model (Gaussian noise), each panel representing one of four conditions (A = Baseline, B = Filtering 1, C = Filtering 2, and D = Filtering 3)**.

Figure 6 contains the Vincentized RT density distributions from the predictions of the RP and PN models. Results with these neurobehavioral models mirror those obtained with their parameterized counterparts: Fits of the RP model with observed data were relatively good [Baseline, χ^2^_(17)_ = 3.91; Filtering 1, χ^2^_(17)_ = 11.85; Filtering 2, χ^2^_(17)_ = 11.96; Filtering 3, χ^2^_(17)_ = 6.65], whereas those of the PN model aligned with observed RTs only in the baseline condition [Baseline, χ^2^_(17)_ = 5.12; Filtering 1, χ^2^_(17)_ = 34.16, *p* < 0.01; Filtering 2, χ^2^_(17)_ = 31.52, *p* < 0.05; Filtering 3, χ^2^_(17)_ = 41.91, *p* < 0.01]. Inspection of distributional moments (Table 4) indicated, once again, that the excitation-based (PN) model was poor during filtering at reproducing measures of central tendency and variability (except in Filtering 3; mean RT underestimated by 31 ms, 31 ms, and 26 ms in the Filtering 1, Filtering 2, and Filtering 3 conditions; variance in RT by 34 ms, 35 ms, and 1 ms). The RP model, on the other hand, accurately reproduced all moments, with the exception of variance in the Filtering 1 (−19 ms) and Filtering 2 (−14 ms) conditions, reminiscent of the inhibition-only model (cf. Table 3). As we shall see, underestimation of RT variance in the two inhibition models may owe to properties of the underlying (Gaussian) noise distribution. In any case, it is worth noting that the RP model, with only 7 free parameters, was at least as good in capturing the RT distributions as the inhibitory-only model with 9 free parameters, and better than the PN model, also with 9 free parameters. This outcome illustrates that actual RP voltage is viable in predicting specific properties of RTs in auditory selective attention tasks, suggesting that RP activity serves as a brain biomarker of processes of distractor inhibition. Still, ANOVA uncovered statistically significant differences between observed and predicted moment values in both the PN and RP models [*PN*: *F*_(1, 10)_ = 6.12, *p* < 0.05; *RP*: *F*_(1, 10)_ = 25.31, *p* < 0.001].

**Table 4:** **Difference between predicted and observed moments in RTs (mean, variance, skewness, and kurtosis) for the RP Model and the PN Model**.

**Condition**	**Mean**	**Variance**	**Skewness**	**Kurtosis**
**RP MODEL (GAUSSIAN NOISE)**				
Baseline	−0.164	−0.543	−0.012	0.282
Filtering 1	−1.884	−16.056	−0.176	−0.253
Filtering 2	−1.536	−14.241	0.121	0.847
Filtering 3	−5.960	−7.300	−0.003	0.214
**PN MODEL (GAUSSIAN NOISE)**				
Baseline	0.001	−2.038	−0.109	0.051
Filtering 1	−31.380	−33.911	−0.089	0.302
Filtering 2	−31.083	−35.336	0.149	0.788
Filtering 3	−26.222	1.102	0.896	7.116

### Random noise vs. EEG noise

Each of the foregoing simulations employed Gaussian noise—computer-generated random numbers from a standard normal distribution—to induce change in RT from one trial to the next. The third set of simulations aimed to evaluate the linking hypothesis that momentary distractor-evoked fluctuations in RP voltage are meaningfully associated with stochastic behavioral processes. We evaluated this hypothesis by comparing the initial inhibition-only model using Gaussian-noise with a comparable model that predicted behavioral variation using RP-noise. An additional simulation implemented a third variant of the inhibition-only model, one that generated RT variability from Rectangular-noise. An RP distribution was created for each participant in each condition using a single-trial EEG analysis (see Parra et al., 2002; Philiastides and Sajda, 2006) in which the amplitude in microvolts of RP at Fz 400–600 ms after stimulus onset was obtained to each distractor trial in which the participant correctly withheld a key press and rescaled by multiplying a constant. In extracting single-trial EEG signals to solitary distractors we eschew any inherent physiological information about response processing, such as the motor activity to targets.

**Figure 7:**
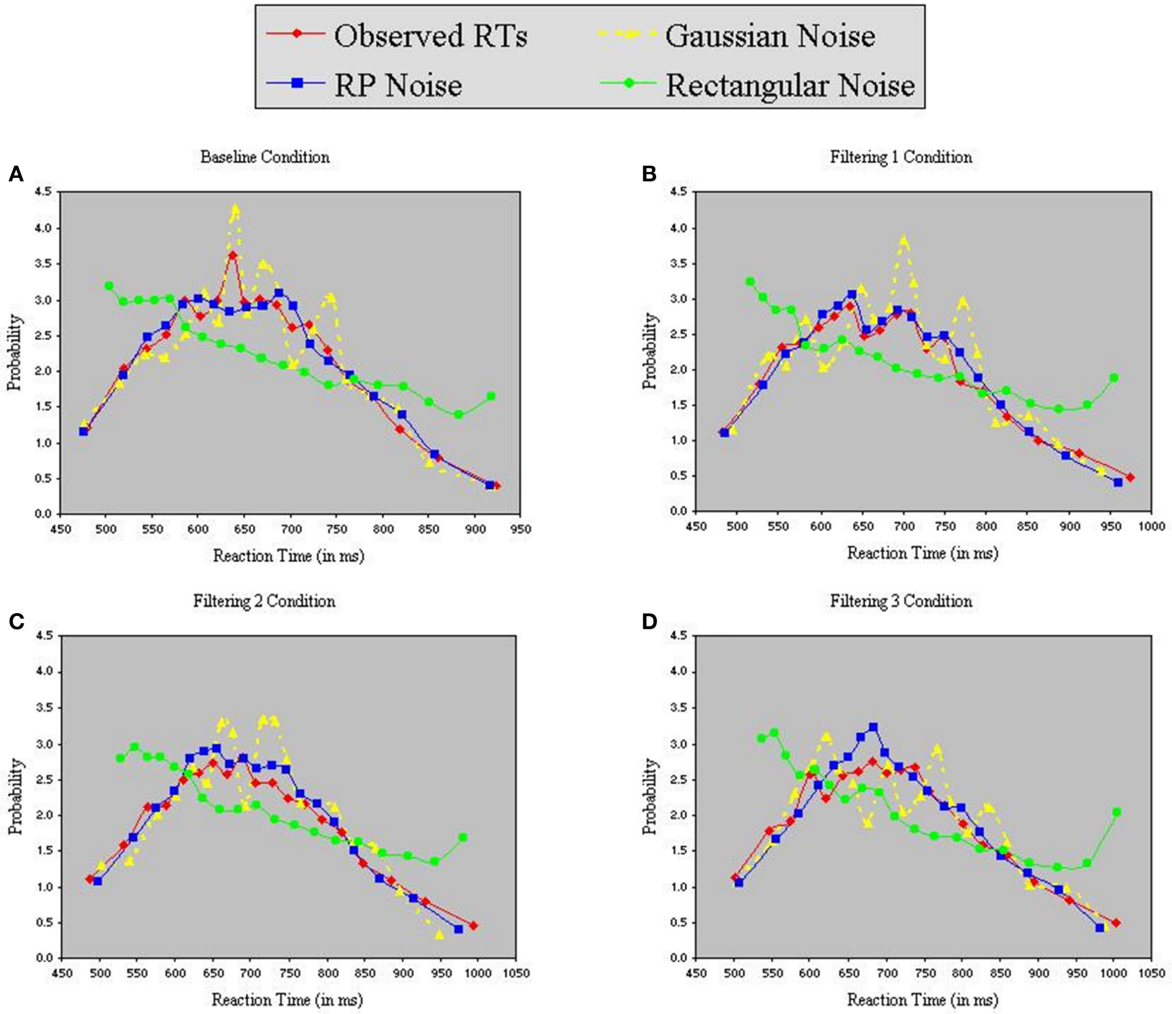
**Probability density functions of observed RTs together with predicted RTs for the Inhibition-Only Model using three methods for generating inter-trial variability: Gaussian noise, RP noise, and Rectangular Noise.** Each panel represents one of four conditions (**A** = Baseline, **B** = Filtering 1, **C** = Filtering 2, and **D** = Filtering 3).

Each model contained 9 free parameters. In each of the three simulations, RTs were sorted from shortest to longest for each participant in each condition, without regard to the particular target (T_1_, T_2_, or T_3_) presented on a trial. A set of *n* values from each of the three distributions (Gaussian, RP, and Rectangular) was selected and sorted from smallest to largest. The backfit solution procedure was then applied such that, for each trial, one of the *n* distributional values served as the value of *x* in Equations 6 and 7, with the smallest random number assigned as *x* to the trial having the shortest RT, and so on through the set. Density functions of predicted RTs from each model appear with observed RTs in the panels of Figure 7. The commonly used Gaussian-noise model, as already noted, underestimated RT variance in two of the three filtering tasks. One can see in Figure 7 that this model was particularly lackluster in simulating RTs in the 600–800 ms range. The Rectangular-noise model, however, proved even worse in fitting the observed data in each of the four conditions [Baseline, χ^2^_(17)_ = 43.11, *p* < 0.01; Filtering 1, χ^2^_(17)_ = 45.92, *p* < 0.01; Filtering 2, χ^2^_(17)_ = 37.66, *p* < 0.01; Filtering 3, χ^2^_(17)_ = 22.13, *ns*]. Predicted RT distributions created from Rectangular-noise were too platykurtic and skewed excessively toward the positive tail (see Table 5). Interestingly, we found that a model using fluctuations in EEG as the source of trial-to-trial variation in RT yielded the best fit. Simulated RTs from the RP-noise model quite accurately mirrored observed RT variance (together with the other three moments) across conditions. In fact, ANOVA revealed no significant differences between observed and predicted moment values in the RP-noise model [*F*_(1, 10)_ = 1.47, *ns*], and no interactions of Simulation with Moment [*F*_(3, 30)_ = 2.29, *ns*], Condition [*F*_(3, 30)_ = 0.59, *ns*], or both [*F*_(9, 90)_ = 0.95, *ns*]. This finding suggests that momentary RP activity to distractors is closely associated with trial-to-trial variability in speed of responding to targets.

**Table 5:** **Difference between predicted and observed moments in RTs (mean, variance, skewness, and kurtosis) for theGaussian-noise, RP-noise, and Rectangular-noise simulations of the Inhibition-Only Model**.

**Condition**	**Mean**	**Variance**	**Skewness**	**Kurtosis**
**GAUSSIAN NOISE**				
Baseline	−0.164	−0.543	−0.012	0.282
Filtering 1	−1.884	−16.056	−0.176	−0.253
Filtering 2	−1.717	−12.208	0.102	0.688
Filtering 3	0.365	−2.351	−0.017	0.088
**RP NOISE**				
Baseline	−0.223	−0.848	−0.038	−0.007
Filtering 1	0.157	−3.274	0.111	0.592
Filtering 2	−0.525	−7.011	0.114	0.574
Filtering 3	−0.192	−5.820	0.088	0.484
**RECTANGULAR NOISE**				
Baseline	1.415	−5.574	−0.516	−1.809
Filtering 1	1.378	−9.102	−0.415	−1.383
Filtering 2	0.688	−10.003	−0.305	−1.178
Filtering 3	1.912	−2.711	−0.245	−1.088

Why did RP-noise provide a better fit than Gaussian-noise (or Rectangular-noise)? We examined standardized probability distributions of RP-noise, Gaussian-noise, and observed RTs in each condition. The observed data more consistently resembled the RP-noise distributions in both variance and skewness across conditions, with distributional co-variation slightly higher between RT and RP-noise probabilities (accounting for 96% of the variance) than between RT and Gaussian probabilities (94% of variance). This slender advantage was enough to give the RP-noise simulations the edge in predicting individual RTs on a trial-by-trial basis.

### Fully linked simulation

We found in the previous set of simulations that a model implementing RP-noise provided a closer match to observed RTs than one implementing Gaussian-noise. Earlier simulations showed that models that modulate distractor inhibition—in the form of either the inhibition-only model or the RP model—offered better fits than models that varied target excitation. In the final pair of simulations we incorporated RP-noise into the two models that modulate distractor inhibition, namely, (1) an inhibition-only version that estimates *dstart* using only free parameters and (2) an RP version that estimates *dstart* using each participant's signal-averaged RP component. The aim of these simulations was to assess the viability of a fully neurobehavioral model: the RP model with RP-noise. We expected that this model, with 7 parameters, would fit the data as well as the inhibition-only model with RP noise, with 9 parameters, our best model to date. This comparison thus provides an especially strong test of the linking hypothesis under study.

**Figure 8:**
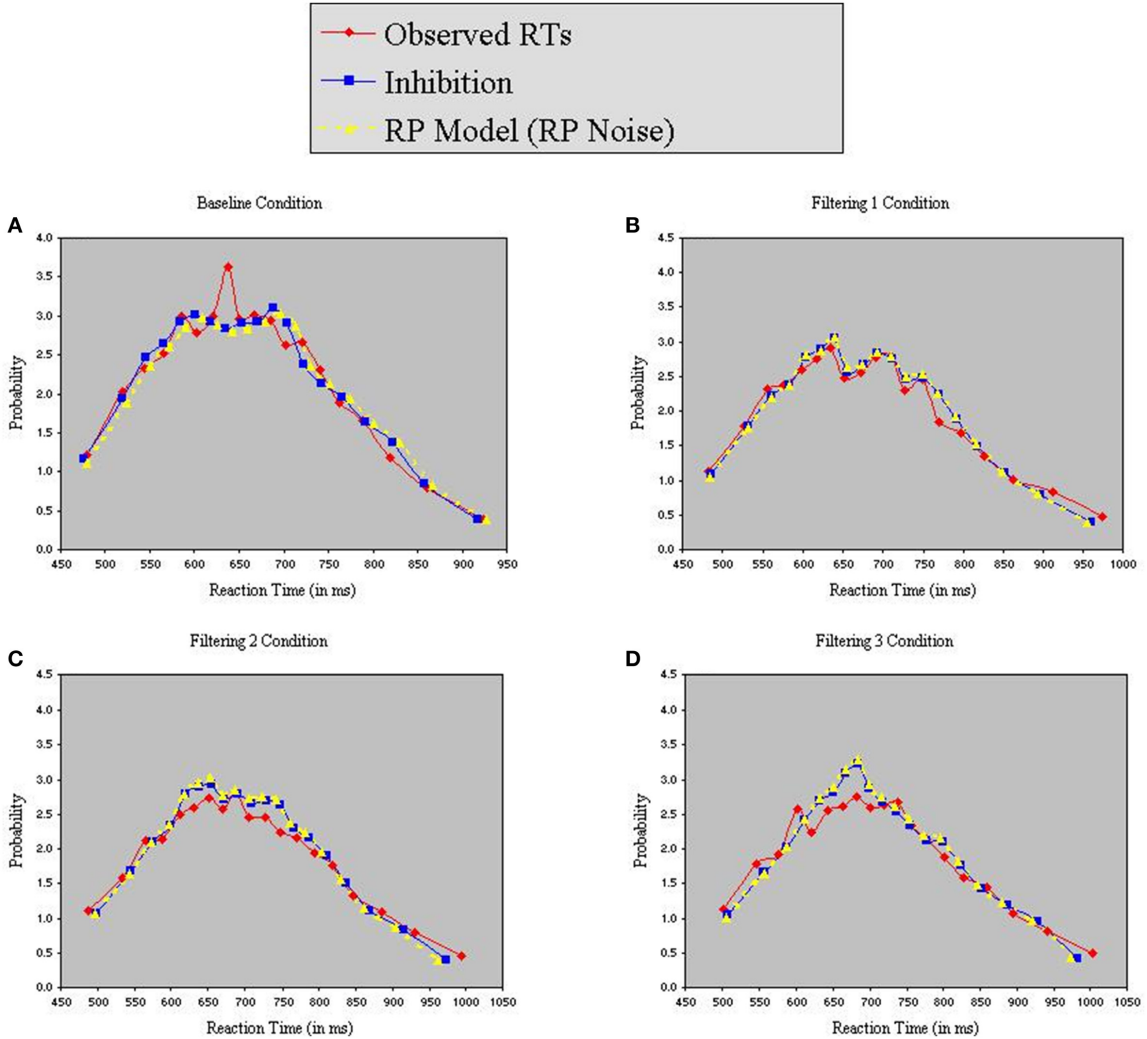
**Probability density functions of observed RTs together with predicted RTs for the Inhibition-Only Model (RP noise) and the RP Model (RP noise), each panel representing one of four conditions (A = Baseline, B = Filtering 1, C = Filtering 2, and D = Filtering 3)**.

Figure 8 reveals that both models provided excellent fits to the observed data. Analysis of distributional moments, depicted in Table 6, confirms that the models captured key distributional properties of the data, particularly with regard to variability and skewness, with neither model showing significant differences between observed and predicted moment values [*Inhibition-Only*: *F*_(1, 10)_ = 1.47, *ns*; *RP*: *F*_(1, 10)_ = 3.51, *ns*]. A common feature of the two models was the slight overestimation of peakedness during performance of the filtering tasks, creating predicted distributions more leptokurtic than the observed distributions (see Table 6). Nevertheless, the correlation between observed and predicted RTs exceeded 0.98 for both models. Thus, with two fewer free parameters, the RP model with RP noise was as good as the inhibition-only model with RP noise at characterizing the observed RT distributions.

**Table 6:** **Difference between predicted and observed moments in RTs (mean, variance, skewness, and kurtosis) for the Inhibition-Only Model with RP Noise and the RP Model with RP Noise**.

**Condition**	**Mean**	**Variance**	**Skewness**	**Kurtosis**
**INHIBITION-ONLY MODEL (RP NOISE)**				
Baseline	−0.223	−0.848	−0.038	−0.007
Filtering 1	0.157	−3.274	0.111	0.592
Filtering 2	−0.525	−7.011	0.114	0.574
Filtering 3	−0.192	−5.820	0.088	0.484
**RP MODEL (RP NOISE)**				
Baseline	7.253	1.018	−0.055	−0.002
Filtering 1	−1.074	−4.641	0.115	0.707
Filtering 2	−5.741	−10.161	0.111	0.657
Filtering 3	−2.928	−9.387	0.079	0.544

## Discussion

Four sets of computer simulations using variants of a formal model of selective attention (Melara and Algom, 2003) probed the role of RP in the inhibitory control of distraction. The simulations used behavioral and electrophysiological data recorded in an earlier study where the degree of distractor salience varied as participants performed an auditory selective attention task involving asynchronous presentation of targets and distractors (Melara et al., 2005). Each simulation sought to predict RTs to targets in the face of different degrees of distraction. The first pair of simulations compared predictions of an inhibition-only vs. excitation-only version of the model, in each case using free parameters to set levels across conditions of inhibitory or excitatory activity, respectively. We found that the inhibition-only model alone accounted satisfactorily for detailed features of the observed RT distributions as distractor salience changed. A second pair of simulations implemented two neurobehavioral versions of the model: an RP model, which incorporated each participant's averaged slow-wave activity to distractors in estimating slopes of distractor inhibition, vs. a PN model, which incorporated averaged slow-wave activity to targets in estimating slopes of target excitation. The RP model, but not the PN model, captured changes in RT performance closely as a function of distractor salience.

The third set of simulations explored how well different sources of stochastic variation predicted individual RTs. One source was the momentary fluctuations in slow-wave RP activity (RP-noise) measured using single-trial EEG analysis. We compared RP-noise with Gaussian-noise and Rectangular-noise, with values drawn randomly from computer-generated normal and rectangular distributions, respectively. We found that RP-noise mimicked inter-trial RT variation more closely than Gaussian-noise and, especially, Rectangular-noise. Finally, in a fully linked simulation, we showed that a version of the RP model that included RP-noise provided an exceptionally good fit to the behavioral data, with the fewest free parameters. Our results are consistent with the hypothesis that RP activity influences the speed of responding to targets during selective attention through the inhibitory modulation of distractors.

### Computational links between physiology and behavior

Melara et al. (2005) found that behavioral performance to targets changed systematically as a function of distractor salience, and was accompanied by a progressive change in RP amplitude to distractors, but not in PN amplitude to targets. The authors concluded that RP serves to suppress intrusions from previously presented distractors as participants make decisions about currently perceived targets. However, average RP amplitude was merely correlated in their study with levels of distractor salience. Thus, it is conceivable that RP functions simply to monitor stimulus salience, playing no causal role in distractor inhibition. The primary contribution of the current study is in showing that the magnitude of RP to distractors closely predicts an individual's RT to targets through modulation of inhibitory control. We therefore were able to link RP explicitly to behavior in the context of a formal computational model of distractor processing. Equally important, we found that PN to targets was relatively ineffective in predicting RTs through the modulation of target activity. Thus, here at least, distractor processing (RP) was more diagnostic of target behavior during selective attention than target processing itself (PN). Later we discuss possible reasons for this result.

The current study focused on a single behavioral measure: RT. A host of recent electrophysiological studies have pointed to the connection between response latency and the amplitude of various ERP components, including N1 (e.g., Melara et al., 2005), P2 (e.g., Sheehan et al., 2005; Tong et al., 2009), MMN (e.g., Atienza et al., 2002), and P3b (e.g., Alain et al., 2007). Ours is among relatively few studies (e.g., Holroyd and Coles, 2002) able to demonstrate the link between EEG amplitude and microscopic changes in RT. Still, other EEG measures, including Nd onset latency and power in EEG bands, and other behavioral measures, including target misses and distractor false alarms, are sensitive to attention manipulations, such as distractor salience. Indeed, in the study that served as the empirical basis of the current simulations, Melara et al. (2005) identified several connections between physiology and behavior. The goal of the current study was to concentrate on a single linking hypothesis. Future studies should aim to relate internal processes to other response measures, including target accuracy, false alarms, misses, and incorrect identifications.

### Representing stochastic behavioral processes using neurophysiological activity

A further contribution of the current study was in demonstrating the feasibility of incorporating momentary fluctuations in neurophysiological activity into a formal model to predict trial-level performance during selective attention. Our simulations using RP-noise revealed that electroencephalographic measurements excelled over random Gaussian or rectangular activity in capturing trial-to-trial variations in RT to targets. Indeed, a fully neurobehavioral version of the model (RP model with RP-noise) was able to account successfully for the RTs of every participant on every trial in every condition of the study.

To be sure, the fidelity of RT prediction from random noise was perforce determined by statistical characteristics of the computer-generated distributions we implemented. Had the stochastic processes been modeled using a probability distribution with a slightly different skewness and kurtosis the results perhaps would imitate those with RP-noise. Our aim was not to abandon the use of random noise distributions in modeling stochastic behavioral processes. Nevertheless, the success of RP-noise in predicting RT variability in the current study serves, at the very least, as proof of concept that nuanced behavioral performance during selective attention can be linked closely to internal neurophysiological events.

Moreover, our results hold potential theoretical significance in suggesting that RP-noise involves activation of distractor representations that, ultimately, carry behavioral consequences. On this view, variability in responding to a currently presented target is due in part to the moment-to-moment changes in activation of recently presented distractors, presumably held in working memory. Fluctuations in distractor activation contribute to the uncertainty inherent on target trials, captured in the model as trial-to-trial shifts in the rates of activation to both targets (slope_T1_, slope_T2_, slope_T3_; see Equation 6) and distractors (slope_d_; see Equation 7). In this context, one might characterize RP-noise as reflecting the momentary success or failure of endogenous attentional processes (such as inhibitory control) in suppressing distractor representations while maintaining representations of target relevance.

### Comparison with other attention theories: biased competition and attentional trace theory

The biased competition model (Desimone and Duncan, 1995; Reynolds et al., 1999) has been the theory of choice in explaining evidence of attentional modulation using single- and multi-unit recordings of neurons in primary and secondary areas of visual cortex (see Reynolds and Chelazzi, 2004, for a review). A canonical paradigm for these studies is visual search, in which animals peruse a field of images in search of a target object (e.g., Moore and Fallah, 2001, 2004; Bichot et al., 2005; Hayden and Gallant, 2005). The model predicts that representations of the target object in working memory bias the competition among items within the receptive field to favor cortical cells responsive to the spatial location of the target, perhaps through gain control. The closer the match between the target representation and an item within the receptive field (or, conversely, the stronger the mismatch with other competing items) the greater the competitive edge the item enjoys in activating spatially corresponding cells in visual cortex.

Simulations in the current study relied on a different model of attention: the tectonic theory of Melara and Algom (2003). Tectonic theory also emphasizes competition in working memory between target and distractor representations as the basis of attentional control. However, in contrast to the biased competition model, the theory discounts target-distractor similarity as the primary source of competition, and space-based modulation as the primary mechanism of attentional control (see also Treue and Martínez-Trujillo, 1999). Instead, in tectonic theory the chief threats to inhibitory control are distractor salience and stimulus uncertainty. On this view, representational variability in targets or distractors, rather than representational comparison processes pitting targets against distractors, best characterizes the dynamic relationship between attention and working memory. In fact, Melara et al. (2005) used their empirical results to claim that representational variability (tectonic) dominates target-distractor similarity (biased competition) in undermining selective attention. The current results go further in demonstrating that control mechanisms lawfully govern distractor variability in working memory at both the individual-trial level and the condition level.

Why is biased competition a less satisfactory account here compared with its other applications? One possibility considers the specific processing demands of the attention paradigm employed. Kahneman and Treisman (1984) divided the selection paradigms most commonly used in attention research into two categories: filtering and selective set. In filtering paradigms, including the dual-channel task used by Melara et al. (2005), participants are asked to focus on signals from a task-relevant channel (e.g., auditory frequencies of a certain timbre) while disregarding signals from a task-irrelevant channel (e.g., frequencies of a different timbre). In selective-set paradigms, including visual search (e.g., Chelazzi et al., 2001; Bichot et al., 2005), participants anticipate or must locate one stimulus among several possible other stimuli. The two paradigms impose different information processing burdens on working memory. During selective set, working memory acts as a convenient hub for testing the degree of alignment between an expected or sought-after stimulus and one currently within gaze or awareness. Here, a premium is placed on mechanisms of comparison, so distractors that resemble those expected or searched should impair processing efficiency. By contrast, during filtering, working memory acts as a sorting center to separate signal (task relevant) from noise (task irrelevant). Here, a premium is placed on mechanisms of suppression, so distractors high in noise (variability) should impair processing efficiency. On this view, models of target-distractor similarity, such as biased competition, would emerge most naturally from results obtained with selective-set paradigms, whereas models of representational variability, such as tectonic theory, would emerge from results obtained with filtering paradigms.

One notable counterexample is Näätänen's (1982, 1992) attentional trace theory, which holds that selective attention performance in filtering paradigms is determined by how closely stimuli in a task-irrelevant channel match the trace in working memory representing stimuli in the task-relevant channel. The closer the match, the larger is the amplitude of PN, indicating greater filtering task difficulty. In this way, attentional trace theory uses target-distractor similarity during filtering to explain competition in working memory between task-relevant and task-irrelevant signals. Here one must distinguish, however, between two types of target-distractor similarity: within channel and between channel (see Melara, 1992). Attentional trace theory focuses on between-channel similarity: PN is greater the smaller the physical separation between signals defined as task relevant vs. task irrelevant (e.g., greater PN for 50 Hz separation between channels than 400 Hz separation; see Hansen and Hillyard, 1983). Melara et al. (2005), on the other hand, manipulated within-channel similarity: Targets defined by one dimension (e.g., timbre) could be more or less similar to distractors along the dimension requiring identification (e.g., pitch).

Using this distinction, one might reason that mechanisms of comparison operate in attention systems whenever similarity *between* target and distractor channels must be resolved—common in selective-set paradigms—whereas mechanisms of suppression operate in attention systems whenever representational variability *within* distractor channels must be controlled—common in filtering paradigms. In line with this reasoning, we found in this study of within-channel filtering that RP, indicative of distractor suppression, was highly predictive of individual RTs to targets whereas PN, indicative of target-distractor comparison, was not. We might expect the fit of PN neurobehavioral models to improve when simulating paradigms where the similarity between target and distractor channels is varied systematically, whether employing selective-set or filtering paradigms.

## Conclusions

Computational analysis revealed that modulation of distractor inhibition using RP voltage accurately predicts the speed of target responding as a function of distractor salience. Our analysis further revealed that oscillations in RP account well for trial-to-trial variations in target performance. In using actual neural activity to computationally model inhibitory control between trials and between tasks, the current study establishes a solid link between RP magnitude and RT performance during selective attention.

### Conflict of interest statement

The authors declare that the research was conducted in the absence of any commercial or financial relationships that could be construed as a potential conflict of interest.

## Footnotes

^1^It is possible that some distractors were attentionally more disruptive than others. Yet in the original empirical experiment (Melara et al., 2005, Experiment 1), the main source of performance errors was within-target misidentifications (e.g., confusing a high-pitched target with a middle-pitched target), not errors of commission (falsely identifying a distractor as a target). The false alarm rate was less than 5% across conditions (though slightly higher, 8%, in Filtering 3), and did not differ by distractor stimulus within condition. By contrast, target misidentifications were over twice this rate. For this reason, and also to reduce modeling complexity, in each filtering condition we simulated the joint effects of the three distractors.
